# Breast Cancer in the United States: A Cross-Sectional Overview

**DOI:** 10.1155/2020/6387378

**Published:** 2020-10-30

**Authors:** Nadeem Bilani, Emily C. Zabor, Leah Elson, Elizabeth B. Elimimian, Zeina Nahleh

**Affiliations:** ^1^Department of Hematology/Oncology, Maroone Cancer Center, Cleveland Clinic Florida, USA; ^2^Department of Quantitative Health Sciences & Taussig Cancer Institute, Cleveland Clinic, USA

## Abstract

**Introduction:**

Breast cancer remains the most commonly diagnosed malignancy in women. It encompasses considerable heterogeneity in pathology, patient clinical characteristics, and outcome. This study describes factors associated with overall survival (OS) of breast cancer in an updated national database.

**Methods:**

We conducted a retrospective analysis of patients with breast cancer diagnosed between 2004 and 2016 based on the National Cancer Database. Categorical variables were summarized using frequencies/percentages, whereas continuous variables were summarized using the median/interquartile range (IQR). OS was explored using the Kaplan-Meier method.

**Results:**

Data from *n* = 2,671,549 patients were analyzed. The median age at diagnosis was 61 years (range 18-90). 75% were non-Hispanic (NH) White; 11% were NH-Black; 4.7% were Hispanic-White; 0.1% were Hispanic-Black; and 3.4% were Asian. Most cases (73%) presented with ductal carcinoma histology; while 15% with lobular carcinoma. Rarer subtypes included epithelial-myoepithelial, fibroepithelial, metaplastic, and mesenchymal tumors. OS was associated with molecular subtype, histologic subtype, and AJCC clinical staging. Survival also correlated with race: a cohort including Asians and Pacific Islanders had the best survival, while Black patients had the worst. Finally, facility type also impacted outcome: patients at academic centers had the best survival, while those at community cancer programs had the worst.

**Conclusion:**

This large database provides a recent and comprehensive overview of breast cancer over 12 years. Molecular subtype, histologic subtype, stage, race, and facility type were correlated with OS. In addition to the educational perspective of this overview, significant factors impacting the outcome identified here should be considered in future cancer research on disparities.

## 1. Introduction

Breast cancer (BC) is the most common cancer in women worldwide and is second to lung cancer as the biggest cancer-related killer in developed countries [1]. While the incidence of female BC continues to rise annually, most commonly driven by hormone receptor-positive, nonmetastatic disease, the mortality rate has dropped around 40% from 1989 to 2017 [1].

The American Cancer Society estimates that in early 2019, there were around 4 million women with a history of BC living in the United States [1]. In this large population of individuals with BC, there is considerable heterogeneity in demographic, clinical, and pathological disease characteristics. This study aims at providing an overview of BC characteristics and prognostic factors in over 2.6 million patients with breast cancers diagnosed between 2004 and 2016 from the National Cancer Database (NCDB).

## 2. Methods

### 2.1. Patient Data

We conducted a retrospective analysis of breast cancer data extrapolated from the National Cancer Database (NCDB) registry. The NCDB is a United States-based, nationwide repository for cancer cases, contributed to by over 1400 Commission on Cancer- (CoC-) and American College of Surgeon- (ACS-) sanctioned facilities. It is estimated to include 70% of all cancer cases in the United States [2]. Hospitals participating in the NCDB contributed the de-identified data used in this study, which was accessed based on a grant award. All contributing institutions collect patient data prospectively and are required to observe the quality practices for accurate documentation of prognostic data, treatment, and patient outcomes [3]. The data were accessed on a Participant User File (PUF) based award, and this study was approved by the Cleveland Clinic Institutional Review Board. Records from patients with American Joint Committee on Cancer (AJCC) clinical stages I-IV breast cancer, diagnosed between 2004 and 2016, were identified within the NCDB data set.

### 2.2. Statistical Analysis

Categorical variables were summarized using frequencies and percentages, whereas continuous variables were summarized using the median and interquartile range (IQR). Overall survival time was calculated from the date of diagnosis to the date of death, and patients still alive were censored at their date of last contact. The Kaplan-Meier method estimated the overall survival probability. The log-rank test was used to compare groups in analyses stratified according to disease stage. Survival was also evaluated by receptor subtype, histologic subtype, race, and facility type. All statistical analyses were conducted using R software version 3.6.1 (R Core Development Team, Vienna, Austria).

## 3. Results

This analysis included *n* = 2,671,529 patients diagnosed with BC between the years 2004 and 2016. Sociodemographic characteristics are summarized in Table 1. The median age at diagnosis was 61 years (range 18-90). The majority of the patients were non-Hispanic (NH) White (*n* = 1,986,450, 75%), *n* = 286,176 (11%) were NH-Black; *n* = 124,877 (4.7%) were Hispanic-White, *n* = 2,977 (0.1%) were Hispanic-Black, and *n* = 90,484 (3.4%) were Asian. The remainder of the cases fell into other categories or had unknown race data. Patients were most likely to have private insurance (*n* = 1,407,397, 54%), followed by Medicare (*n* = 982,281, 37%), and Medicaid or other governmental insurance (*n* = 178,762, 6.8%). However, *n* = 52,816 (2.0%) were uninsured. The majority of patients (45%) received therapy at comprehensive community cancer programs (CPs) (*n* = 1,154,278), followed by 30% (*n* = 776, 409) at academic/research CPs, 15% (*n* = 376, 928) at integrated network CPs and 9.5% (*n* = 243,300) at community CPs. Furthermore, most patients (56%) were treated at institutions located within 10 miles of their place of residence (*n* = 1, 488,936), and only 6.9% (*n* = 182,487) traveled more than 50 miles from their place of residence to their treatment center.

Clinical characteristics are summarized in Table 2. The most prevalent subtype of BC was hormone receptor-positive (either estrogen receptor or progesterone receptor) and HER2 negative: (HR+)/HER2- (58%, *n* = 583, 113), followed by HR+/HER2+ (26%, *n* = 258,750), HR-/HER2-negative (10%, *n* = 104,175), and HR-/HER2+ (6.4%, *n* = 64, 947). Most patients were diagnosed at stage I (42%, *n* = 1,070,218), followed by stage II (25%, *n* = 647,845), stage 0 (21%, *n* = 534,244), stage III (8.6%, *n* = 220,878), and stage IV (4.0%, *n* = 102,954) of disease.

In this cohort, 73% of cases had ductal carcinoma (*n* = 1, 957,275) and 15% lobular carcinoma (*n* = 402, 325). The remaining histological subtypes included <0.1% adenocarcinoma of mixed types (*n* = 1669), 0.7% intraductal papillary (*n* = 19,330), 0.4% papillary (*n* = 9,536), 0.9% epithelial-myoepithelial (*n* = 23,751), 0.1% fibroepithelial (*n* = 3,498), 0.4% metaplastic (*n* = 11,629), <0.1% mesenchymal (*n* = 1, 758), <0.1% tumors of the nipple (*n* = 1,736), <0.1% carcinoid tumors (*n* = 64), <0.1% malignant lymphomas (*n* = 20), 0.3% inflammatory invasive carcinoma (*n* = 8,803), 1.6% rare breast carcinomas (*n* = 43,787), and 7% other carcinomas (*n* = 186,368). Tumor grade was well/moderately-differentiated in 1,535,058 cases (65%), poorly-differentiated in 792,145 cases (34%), and undifferentiated in 20,949 (0.9%). Among patients diagnosed at stage IV, bone as the most common metastatic site (*n* = 6,934, 68%); followed by lung (*n* = 3,072, 31%), liver (*n* = 2,476, 25%), and brain (*n* = 793, 7.9%) involvement, though the majority of cases of stage IV did not document the location of metastatic sites. Most patients received hormonal therapy (87%, *n* = 1,475,279), while 35% (*n* = 907,505) received chemotherapy, and 3.6% (*n* = 94,220) received immunotherapy. Radiotherapy was used in 53% of patients (*n* = 1,389,019), while 93% (*n* = 2,471,109) underwent any surgery. Lumpectomy was used more frequently than mastectomy (55% vs. 38%, respectively). Surgical margins were negative for residual disease in the vast majority of patients undergoing surgery (93.5%, *n* = 2,315,928).

**Figure 1:**
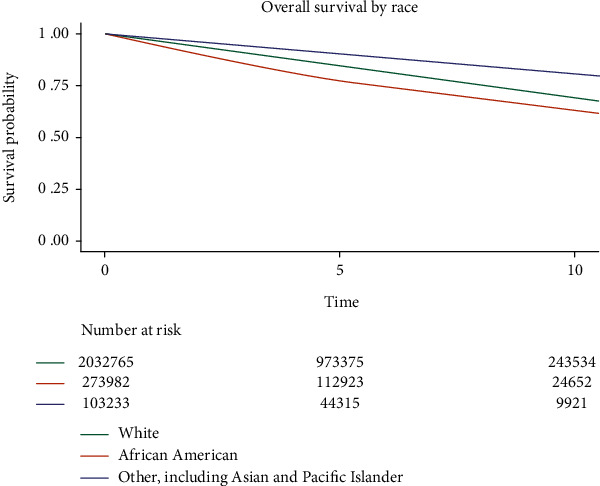
Kaplan-Meier survival curve stratified by race.

**Figure 2:**
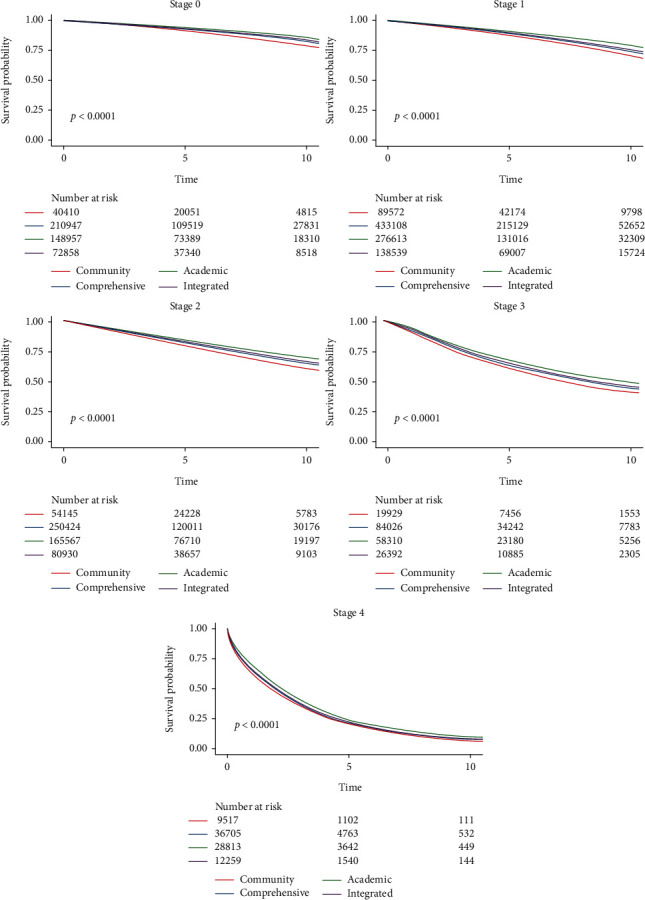
Kaplan-Meier survival curves stratified by facility type and stage.

A total of 2,436,693 patients had available data on *both* vital status and date of death or last contact and were available for analyses of overall survival. Median follow-up among survivors was 4.7 years (IQR: 2.2-7.7 years) and during this time, 456,605 patients died from any cause. 5-year and 10-year overall survival (OS) data are summarized in Table 3. *By stage*: OS progressively declined with disease stage: 5-year OS was 95% for patients diagnosed at stage 0 (10-year OS was 83%), 90% for stage I (10-year OS was 75%), 83% for stage II (10-year OS was 67%), 66% for stage III (10-year OS was 47%), and 23% for stage IV (10-year OS was 9.2%). *By molecular subtype*: OS was highest for patients with HR+/HER2- disease (84% 5-year OS), followed by HR+/HER2+ (83% 5-year OS), HR-/HER2+ (77%), and finally TNBC (71% 5-year OS). 10-year OS could not be assessed by molecular subtype as HER2 status was not documented in the NCDB prior to 2009. *By histologic subtype*: The most common histologic subtype, ductal carcinoma, was associated with an 84% 5-year OS and 70% 10-year OS. Similar survival rates were seen in the next most common subtype, lobular carcinoma (84% 5-year OS, 68% 10-year OS). Patients with rare breast carcinomas had the best survival (92% 5-year OS, 80% 10-year OS), while those with mesenchymal tumors had the worst survival (47% 5-year OS, 35% 10-year OS). *By race*: OS was better for White patients (84% 5-year OS and 69% 10-year OS versus 78% 5-year OS and 63% 10-year OS in Black patients). The best overall OS was noted in the third cohort of patients, which included Asians and Pacific Islanders (90% 5-year OS and 81% 10-year OS). This disparity in survival by race is presented in Figure 1. *By facility type*: OS was noted to be the highest at academic centers (85% 5-year OS and 72% 10-year OS), followed by at integrated network (84% 5-year OS and 69% 10-year OS), then comprehensive community (83% 5-year OS and 68% 10-year OS) and community (80% 5-year OS and 63% 10-year OS) CPs. Significant differences in OS according to facility type were present when stratified by stage of disease (all *p* < 0.0001). This is presented in Figure 2.

## 4. Discussion

This large dataset from the NCDB provides a comprehensive, recent overview of clinicopathologic and sociodemographic factors in patients with BC, spanning over more than a decade.

The most prevalent molecular subtype of BC continues to be HR+/HER2-negative, followed, interestingly, by HR+/HER2-positive, HR-/HER2-negative, and HR/HER2+ disease (Table 2). Molecular biomarkers are primarily determined using immunohistochemical staining methods and serve as the cornerstone of clinical decision-making in BC [5]. With the vast majority of BC cases having HR+ disease, and ER+ or PR+ disease continuing to be the most common type of BC diagnosed [1], it is likely that hormonal–based therapy will remain the mainstay of BC treatment moving forward, including the treatment of HR+/HER-2 positive subtypes in early and advanced stages of BC [6, 7].

However, researchers have also been interested in the extent to which BC histological subtype carries with it prognostic and predictive significance. This large, cross-sectional study outlines a range of atypical histological subtypes (contributing 27% of all BC cases), in addition to invasive lobular carcinomas, other rare tumors, including papillary, epithelial-myoepithelial, metaplastic, and mesenchymal morphologies (Table 2). The rarity of these subtypes has been a limiting factor to research investigating the significance of morphology on outcomes. We found that histological subtype may be associated with disparities in overall survival. This may be due to the relationship between tumor histology and differences in metastatic potential. As an example, patients with metastatic lobular carcinoma are more likely to have disease involvement of the bone, but are less likely to have multiple secondary disease sites at diagnosis, compared to those with ductal carcinoma [8]. Also, emerging evidence has suggested that histology should be included as one of the considerations when determining the most appropriate treatment course [5, 8–12]. Thus far, the *National Comprehensive Cancer Network* has designated the novel categories of “favorable” or “unfavorable” to histological subtypes and recommends triaging optimal management based on this classification [13]. The use of this large registry has helped confirm the prevalence of these tumor types and emphasizes the importance of furthering our knowledge of these less common histological types.

AJCC clinical staging correlated with survival as expected, with the best 5-year OS and 10–year OS noted in patients diagnosed with stage 0 (95% and 83%, respectively). Most patients were diagnosed at early stages (Table 2), which is very encouraging. What is noteworthy is the 5–year OS of 23% for patients with stage IV BC, which was once reported to be as low as 10% for patients diagnosed between 1985 and 1990 [14]. 10-year OS is more scarcely reported [15], but were often not reached in cohort studies before 2000 [16]. This improvement is welcomed news and contributed to by decades of advances in treatment and successful targeted approaches in the field of metastatic breast cancer. The data also suggest that patients with HR+ tumors have the best prognosis, with similar 5-year OS noted for HR+/HER2- and HR+/HER2+ compared to HR/HER2+, followed by HR-/HER2-, which had the worst outcome.

This study reiterates the significant impact of race on both immediate and long-term survival, with Black patients exhibiting the worst survival (Table 3, Figure 1). This finding is consistent with descriptive reports that Black patients are more likely to be uninsured or underinsured [17], have more comorbidities [18], be diagnosed at later stages [1], and experience more treatment delays [18] compared to their White counterparts. Additionally, Black patients are more likely to have triple-negative breast cancer (TNBC) [17, 19], an aggressive subtype of the disease. Interestingly, a cohort including Asians and Pacific Islanders was noted to have the best survival (Table 3, Figure 1). This superior outcome is consistent with data published by the American Cancer Society showing these patients were more likely to be diagnosed at earlier stages of disease [1]. More research is needed to understand the experience of BC in understudied minority groups—including Asians, Pacific Islanders, and Native Americans—and better understand possible lifestyle or biological characteristics correlated with survival. Additionally, more efforts continue to be needed to end racial disparities affecting Black patients with BC, which has continued to exist over several decades.

Finally, this study has also suggested there is a significant relationship between facility type used for treatment and cancer survivorship. OS was best when patients were treated at designated academic centers, followed by integrated network comprehensive community cancer programs, and community cancer programs. This relationship appears to be independent of disease stage, such that academically-designated centers have superior OS across all stages. The disparity was more apparent when considering long-term survival (10-year OS), which is rarely reported in the literature. Previous studies have reported that facility type was significantly associated with time-to-treatment [20, 21] as well as choice of treatment [22] in patients with BC, which may, at least in part, explain our observed differences in survival. More research is needed to better understand potential differences in the patient population, or quality and consistency of care provided based on facility type. Survival analyses in this manuscript are based on univariable testing, so there is a potential for confounding that has not been accounted for that should be explored in future research. Additionally, overall survival metrics reflect mortality due to comorbidities not of primary interest in this manuscript, such as cardiac and lung disease. However, the NCDB does not report cancer-related mortality.

In conclusion, this large research document using the NCDB provides a recent and comprehensive overview of BC over the last 12 years. A favorable prognosis was noted for HR+ tumors regardless of HER2 status. HR-/HER2+ tumors had an inferior prognosis compared to HR+ tumors, but the worst outcome was noted for HR-/HER2+ disease. The latter 2 subtypes can, therefore, be considered high-risk for treatment and surveillance purposes. A notable increase in survival was identified in patients with metastatic breast cancer over the past decade, calling for continuing research efforts in this field. Significant clinicopathological and sociodemographic factors impacting outcomes were identified in this study and could be considered for future research focusing on cancer disparities.

## Figures and Tables

**Table 1:** Sociodemographic characteristics of breast cancer patients diagnosed between 2004 and 2016.

Variable	# (%)
Age (median, range)	61, 51-71
Race/ethnicity	
Non-Hispanic White	1,986,450 (75%)
Non-Hispanic Black	286,716 (11%)
Hispanic White	124,877 (4.7%)
Hispanic Black	2,977 (0.1%)
Asian	90,484 (3.4%)
Other	162,516 (6.1%)
Unknown	17,529
Insurance status	
Uninsured	52,816 (2.0%)
Private insurance	1,407,397 (54%)
Medicaid/other governmental insurance	178,762 (6.8%)
Medicare	982,281 (37%)
Unknown	50,293
Facility type	
Community cancer program	243,300 (9.5%)
Comprehensive community cancer program	1,154,278 (45%)
Academic cancer program	776,409 (30%)
Integrated network cancer program	376,928 (15%)
Unknown	120,634
Great circle distance	
≤10	1,488,936 (56%)
>10-20	584,871 (22%)
>20-30	228,374 (8.6%)
>30-40	112,542 (4.2%)
>40-50	63,913 (2.4%)
>50	182,487 (6.9%)
Unknown	10,426
Setting	
Metro	2,228,924 (86%)
Urban	328,184 (13%)
Rural	42,825 (1.6%)
Unknown	71,616

**Table 2:** Clinical characteristics of breast cancer patients diagnosed between 2004 and 2016.

Variable	# (%)
^∗^Charlson/Deyo comorbidity score	
0	2,259,335 (85%)
1	325,670 (12%)
2	64,289 (2.4%)
≥3	22,255 (0.8%)
Clinical stage at diagnosis	
0	534,244 (21%)
I	1,070,218 (42%)
II	647,845 (25%)
III	220,878 (8.6%)
IV	102,954 (4.0%)
Unknown	95,410
^∗∗^Secondary sites in patients diagnosed at stage IV	
Bone	6,934 (68%)
Brain	793 (7.9%)
Liver	2,476 (25%)
Lung	3,072 (31%)
Molecular subtype	
ER+ or PR+, HER2-	583,113 (58%)
ER+ or PR+, HER2+	258,750 (26%)
ER- and PR-, HER2-	104,175 (10%)
ER- and PR-, HER2+	64,947 (6.4%)
Unknown	1,660,564
Histological subtype	
Ductal carcinoma	1,957,275 (73%)
Lobular carcinoma	402,325 (15%)
Adenocarcinoma of mixed types	1,669 (<0.1%)
Rare breast carcinomas	43,787 (1.6%)
Inflammatory invasive carcinoma	8,803 (0.3%)
Other carcinomas	186,368 (7.0%)
Intraductal papillary	19,330 (0.7%)
Papillary neoplasms	9,536 (0.4%)
Epithelial-myoepithelial	23,751 (0.9%)
Fibroepithelial tumors	3,498 (0.1%)
Metaplastic tumors	11,629 (0.4%)
Mesenchymal tumors	1,758 (<0.1%)
Tumors of the nipple	1,736 (<0.1%)
Carcinoid tumors	64 (<0.1%)
Malignant lymphoma	20 (<0.1%)
Grade	
Well/moderately differentiated	1,535,058 (65%)
Poorly differentiated	792,145 (34%)
Undifferentiated	20,949 (0.9%)
Unknown	323,397
Any hormonal therapy	1,475,279 (87%)
Unknown	978,708
Any chemotherapy	907,505 (35%)
Unknown	89,559
Any immunotherapy	94,220 (3.6%)
Unknown	34,964
Any radiation therapy	1,389,019 (53%)
Unknown	29,641
Type of surgery	
None	194,693 (7.3%)
Lumpectomy	1,459,286 (55%)
Mastectomy	1,007,588 (38%)
Unknown	9,982
Surgical margins	
No surgery	194,693 (7.4%)
No residual disease	2,315,928 (88%)
Residual disease	109,716 (4.2%)
Unknown	51,212

^∗^The Charlson/Deyo index is a weighted score assessing overall comorbidity in patients by cross-referencing these cases to a list of international disease classification (ICD) codes known to be important prognosticators of morbidity and mortality [4]. ^∗∗^The majority of stage IV patients did not have metastatic site documented, thus these percentages are reflective of the total number of cases for which data were available on this variable (*n* = 10,131 bone; *n* = 10,058 brain; *n* = 10,085 liver; *n* = 10,046 lung).

**Table 3:** 5-year and 10-year overall survival (OS) of patients diagnosed between 2004 and 2016.

Variable	5-year OS		10-year OS	
	Probability	95% CI	Probability	95% CI
By stage				
0	94%	94-94%	83%	83-84%
I	90%	90-90%	75%	75-76%
II	83%	83-83%	67%	67-67%
III	66%	66-66%	47%	47-47%
IV	23%	23-23%	9.20%	8.9-9.5%
By molecular subtype^∗^				
HR+, HER2-	84%	84-84%	—	—
HR+, HER2+	83%	83-84%	—	—
HR-, HER2-	71%	71-71%	—	—
HR+, HER2+	77%	76-77%	—	—
By histologic subtype				
Ductal carcinoma	84%	84-84%	70%	70-70%
Lobular carcinoma	84%	84-85%	68%	68-69%
Adenocarcinoma of mixed subtypes	81%	79-84%	65%	61-69%
Metaplastic carcinoma	63%	62-64%	50%	48-51%
Rare breast carcinomas	92%	91-92%	80%	79-81%
Inflammatory invasive carcinoma	39%	38-40%	25%	24-26%
Other carcinomas	84%	84-84%	71%	70-71%
Epithelial-myoepithelial	56%	55-57%	44%	43-45%
Intraductal papillary	90%	89-90%	78%	77-79%
Papillary	83%	82-84%	63%	62-65%
Fibroepithelial	83%	82-85%	74%	71-76%
Mesenchymal	47%	44-50%	35%	31-38%
Tumors of the nipple	81%	79-83%	65%	61-68%
Carcinoid tumor	60%	39-92%	40%	16-99%
Malignant lymphoma^∗∗^	65%	41-100%	—	—
By race				
White	84%	84-84%	69%	69-70%
Black	78%	78-78%	63%	63-63%
Other, including Asian and Pacific Islander	90%	90-90%	81%	80-81%
By facility type				
Community cancer program	80%	80-80%	63%	63-64%
Comprehensive community cancer program	83%	83-83%	68%	68-68%
Academic cancer program	85%	85-85%	72%	72-72%
Integrated cancer program	84%	84-84%	69%	69-70%

^∗^10-year OS data is not available by receptor subtype, as HER2 status is not documented in NCDB prior to 2009. ^∗∗^10-year OS data also not available for patients with malignant lymphoma of the breast due to the year of diagnosis within the last years.

## Data Availability

The data that support the findings of this study are available from the American College of Surgeons/American Cancer Society but restrictions apply to the availability of these data, which were used under license for the current study, and so are not publicly available. Data are however available from the authors upon reasonable request and with permission of the American College of Surgeons/American Cancer Society.

## Additional Points

*Highlights*. There is considerable pathological and clinical heterogeneity in breast cancer. By race: a cohort of Asians and Pacific Islanders had the best survival, while Blacks had the worst. Patients treated at academic centers had the best survival, while those at community cancer programs had the worst. Data on 10-year overall survival in metastatic disease is improved from previously-reported.

## References

[1] DeSantis C. E., Ma J., Gaudet M. M. (2019). Breast cancer statistics, 2019. *CA: a Cancer Journal for Clinicians*.

[2] Bilimoria K. Y., Stewart A. K., Winchester D. P., Ko C. Y. (2008). The National Cancer Data Base: a powerful initiative to improve cancer care in the United States. *Annals of Surgical Oncology*.

[3] American College of Surgeons *National Cancer Database*.

[4] National Cancer Data Base Participant User File (PUF) Data Dictionary. Version: PUF. https://www.facs.org/-/media/files/quality-programs/cancer/ncdb/puf_data_dictionary.ashx.

[5] Bandyopadhyay S., Bluth M. H., Ali-Fehmi R. (2018). Breast carcinoma: updates in molecular profiling 2018. *Clinics in Laboratory Medicine*.

[6] Statler A. B., Hobbs B. P., Wei W., Gupta A., Blake C. N., Nahleh Z. A. (2019). Real-world treatment patterns and outcomes in HR+/HER2+ metastatic breast cancer patients: a National Cancer Database Analysis. *Scientific Reports*.

[7] Statler A. B., Wei W., Gupta A., Blake C. N., Hobbs B. P., Nahleh Z. A. (2020). Elucidating determinants of survival disparities among a real-world cohort of metastatic breast cancer patients: a National Cancer Database Analysis. *Clinical Breast Cancer*.

[8] Lobbezoo D., Truin W., Voogd A. (2016). The role of histological subtype in hormone receptor positive metastatic breast cancer: similar survival but different therapeutic approaches. *Oncotarget*.

[9] Purushotham A., Pinder S., Cariati M., Harries M., Goldhirsch A. (2010). Neoadjuvant chemotherapy: not the best option in estrogen receptor-positive, HER2-negative, invasive classical lobular carcinoma of the breast. *Journal of Clinical Oncology*.

[10] Colleoni M., Rotmensz N., Maisonneuve P. (2012). Outcome of special types of luminal breast cancer. *Annals of Oncology : Official Journal of the European Society for Medical Oncology*.

[11] Akiyama F., Horii R. (2009). Therapeutic strategies for breast cancer based on histological type. *Breast Cancer*.

[12] Singh K., He X., Kalife E. T., Ehdaivand S., Wang Y., Sung C. J. (2018). Relationship of histologic grade and histologic subtype with oncotype Dx recurrence score; retrospective review of 863 breast cancer oncotype Dx results. *Breast Cancer Research and Treatment*.

[13] Breast Cancer National Comprehensive Cancer Network. https://www.nccn.org/professionals/physician_gls/pdf/breast_blocks.pdf.

[14] Dunphy F. R., Spitzer G., Fornoff J. E. (1994). Factors predicting long-term survival for metastatic breast cancer patients treated with high-dose chemotherapy and bone marrow support. *Cancer*.

[15] Soerjomataram I., Louwman M. W. J., Ribot J. G., Roukema J. A., Coebergh J. W. W. (2008). An overview of prognostic factors for long-term survivors of breast cancer. *Breast Cancer Research and Treatment*.

[16] Rhomberg W., Rhomberg T. (2014). Long-term survival in patients with incurable breast cancer. An analysis of 93 cases. *Anticancer Research*.

[17] Akinyemiju T., Moore J. X., Ojesina A. I., Waterbor J. W., Altekruse S. F. (2016). Racial disparities in individual breast cancer outcomes by hormone-receptor subtype, area-level socio-economic status and healthcare resources. *Breast Cancer Research and Treatment*.

[18] Murray D. C. D., Bhandari S., Ngo P. (2019). Race as an independent factor for survival in breast cancer patients according to analysis of the National Cancer Database (NCDB). *American Society of Clinical Oncology*.

[19] Howlader N., Altekruse S. F., Li C. I. (2014). US incidence of breast cancer subtypes defined by joint hormone receptor and HER2 status. *JNCI: Journal of the National Cancer Institute*.

[20] Bhandari S., Ngo P., Mudra S. (2019). Treatment delays in localized breast cancer: A NCDB analysis. *American Society of Clinical Oncology*.

[21] Khorana A. A., Tullio K., Elson P. (2019). Time to initial cancer treatment in the United States and association with survival over time: an observational study. *PLoS One*.

[22] Bagegni N. A., Tao Y., Ademuyiwa F. O. (2019). Clinical outcomes with neoadjuvant versus adjuvant chemotherapy for triple negative breast cancer: a report from the National Cancer Database. *PLoS One*.

